# Effect of High Fat Loading in Dahl Salt-Sensitive Rats

**DOI:** 10.1080/10641960902825487

**Published:** 2009-07-27

**Authors:** Ai Nagae, Megumi Fujita, Hiroo Kawarazaki, Hiromitu Matsui, Katsuyuki Ando, Toshiro Fujita

**Affiliations:** Department of Nephrology and Endocrinology, University of Tokyo Graduate School of Medicine, Tokyo, Japan

**Keywords:** obesity, salt sensitivity of blood pressure, urinary protein, insulin resistance, sympathetic nervous system

## Abstract

Salt sensitivity of blood pressure (BP) is speculated to be a characteristic in obesity-induced hypertension. To elucidate the influence of obesity on salt-sensitive hypertension, we examined the effect of fat loading on BP, renal damage, and their progression induced by salt excess in Dahl salt-sensitive (S) rats. High fat (HF: 45% fat diet: 8 weeks) diet increased BP with greater weight gain and visceral fat accumulation than low fat (10% fat) diet. In HF-fed rats, plasma glucose, plasma insulin, and urinary catecholamine increased, and urinary protein tended to be elevated. Moreover, excessive salt (8% salt diet: 8 weeks)-induced hypertension and proteinuria was accelerated in HF-fed rats. Therefore, fat loading increased BP in Dahl S rats possibly through insulin-resistance and sympathetic excitation. Moreover, fat loading accelerated salt-induced BP elevation and renal damage, suggesting excessive intake of both fat and salt, such as a civilized diet, exert the synergic harmful effects.

## Introduction

It has been believed that obesity is one of the factors that accentuate blood pressure (BP) response to excessive salt (1). Actually, long-term high fat (HF) intake caused greater salt-induced BP rise associated with body weight gain in rats (2). Salt reduction decreased BP more significantly in obese subjects than in nonobese ones and weight loss suppressed a salt-induced rise in BP (3). Thus, there is synergism in the pressor effect between body weight gain and excessive salt. Actually, salt-sensitive hypertension exhibits insulin resistance similarly to hypertension associated with obesity (4). For example, we have demonstrated that salt loading decreased the glucose infusion rate during the hyperinsulinemic euglycemic clamp study and insulin-induced 2-deoxy glucose uptake into isolated soleus muscle in Dahl salt-sensitive (S) rats, an animal model of salt-sensitive hypertension (5). Dahl S rats had a higher plasma insulin concentration than normotensive Splague-Dawley (SD) rats even on a normal salt diet (6). In patients with essential hypertension, moreover, BP rise with salt loading was positively correlated with steady-state plasma glucose during high salt intake (7). As expected, insulin resistance may contribute to hypertension in Dahl S rats, because a high sucrose diet increased BP in Dahl S rats, which was reversed by troglitazone (8). Therefore, insulin resistance may be a common background between obesity- and salt-induced hypertension.

Both obesity and hypertension are risk factors for chronic kidney disease (CKD) (9). Diet-induced obesity causes glomerulosclerosis in C57BL/6J mice (10). As compared to spontaneously hypertensive rats (SHR), an obese hypertensive animal model, SHR/NDmcr-cp accelerated progress of renal damage (11). In addition, Dahl S rats, a model of salt-sensitive hypertension, had more severe renal damage, as compared to SHR, a model of nonsalt-sensitive hypertension, even if their BP levels were the same (12). A recent report has indicated that, in a genetic model of metabolic syndrome, SHR/NDmcr-cp, salt loading extremely exacerbated renal injury (13).

Thus, it is speculated that diet-induced obesity also strengthens excessive salt-induced progression of renal damage as well as a rise in BP. In the present study, in Dahl S rats, we examined the effect of fat loading on BP, renal damage, and their progression induced by salt excess.

## Methods

### Animal Preparation

The present study was conducted in 5-week-old male Dahl S rats, purchased from Japan SLC, Ltd. (Hamamatsu, Japan). They were randomly selected to be fed a HF diet with 0.3% salt (45% kcal as fat, Research Diets, New Brunswick, NJ) for 8 weeks, whereas others were fed a purified low-fat (LF) diet with 0.3% salt (10% kcal as fat). After 8 weeks of the treatment, some animals from both groups were additionally loaded high salt (8.0% salt) for 8 weeks. All rats were housed in a room maintained at 23–25°C with lights on at 7:00 AM and off at 7:00 PM. Rats were given food and water *ad libitum*. Food intake was evaluated by subtracting the remaining food weight from the supplied food weight. All animal procedures conformed to the guiding principles for animal experimentation as enunciated by the Ethics Committees on Animal Research of University of Tokyo Graduate School of Medicine.

### Measurements of BP and Biochemical Parameters

A systolic BP (SBP) measurement was done by the tail-cuff method (P-98A; Softron, Tokyo, Japan). Urine samples were collected for 24 h at 2, 4, 6, and 8 weeks of fat loading and 8 weeks of salt plus fat loading. Urinary sodium was measured by flame photometory. Urinary protein was evaluated using the Bradford method. Urinary creatinine (pure auto SCRE-L, Daiichikagaku Co., Ltd., Tokyo, Japan) and catecholamines (Wakosil-II RS, Wako Pure Chemical Co., Ltd., Tokyo, Japan) were measured by commercial kits.

All rats were fasted overnight and then euthanized with sodium pentobarbital (50mg/kg, intraperitoneally). Blood samples were collected by cardiac puncture at the last day of fat loading. Blood glucose was determined using the Quickauto NEO GLU-HK (Shino-Test Inc., Tokyo, Japan). Plasma insulin and serum leptin were evaluated using radioimmunoassay kits for rat insulin (Linco Research Inc., Saint Charles, MO), and rat leptin (IBL, Gunma, Japan). Total cholesterol and triglycerides were measured by enzymatic methods. Wet weights of total retroperitoneal, epididymal, and mesenteric fats were measured to evaluate visceral fat with standard procedure (14,15) after 4 and 8 weeks of the treatment.

### Renal Histological Evaluation

After fat loading alone and after fat and salt loading, the animals were euthanized by an overdose of pentobarbital. Two kidneys were excised from each group at each point of time, fixed with 4% paraformaldehyde and embedded in paraffin. Sections (3 μm thick) were stained with the periodic acid-Schiff (PAS) method, and examined under light microscopy.

### Statistical Analysis

Data were expressed as means ± SEM. The mean values in the two groups were compared by unpaired *t*-test. Analysis of variance with repeated measurements and subsequent multiple comparison test (Dunnett) were applied to test the effect of treatment on body weight, BP, and urinary protein. Probability values of p < 0.05 were considered to indicate statistical significance.

## Results

### Effects of HF Diet on Body Weight and Visceral Fats

**Figure 1:**
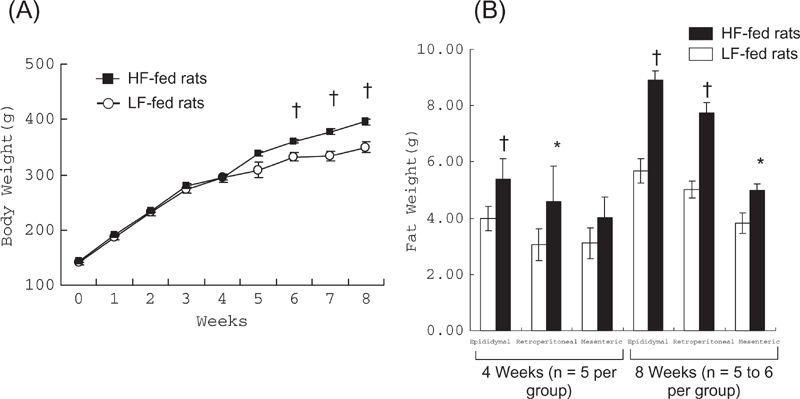
Changes in body weight and visceral fat accumulation with fat loading. (A) After 6 weeks of the treatment, body weight was increased in both groups of rats but its extent was greater in high fat (HF)-fed rats (n = 12) compared to low fat (LF)-fed rats (n = 10). (B) Visceral (retroperitoneal, epididymal, and mesenteric) fat weights were higher in HF-fed rats. *P < 0.05, ^†^P < 0.01 vs. LF-fed rats, respectively.

Body weight was increased during the treatment in both groups of rats, but after 6 weeks of the treatment, it was significantly (P < 0.05) higher in HF-fed Dahl S rats compared with LF-fed rats (Figure 1A). At 8 weeks of the treatment, body weight was approximately 12% greater in HF-fed rats (356.7 ± 9.4 g vs. 400.7 ± 6.4 g, P < 0.05). Visceral fat accumulation preceded body weight gain (Figure 1B); at 4 weeks of the treatment, the visceral fat weight was increased in HF-fed rats, similarly to the data at 8 weeks of the treatment.

### Effects of HF Diet on SBP

**Figure 2:**
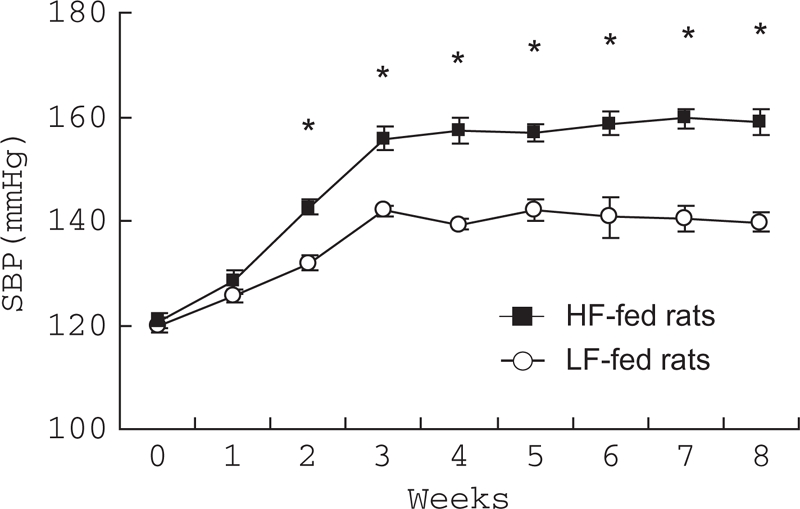
Changes in systolic blood pressure (SBP) with fat loading. SBP was significantly increased in high fat (HF)-fed rats (n = 12) compared with low fat (LF)-fed rats (n = 10) after 2 weeks of treatment. *P < 0.01 vs. LF-fed rats, respectively.

High Fed-fed rats exhibited significantly high SBP after 2 weeks of the treatment (P < 0.01; Figure 2); hypertension also preceded body weight gain as visceral fat accumulation. At the end of the 8-week treatment, SBP was significantly greater in HF-fed rats compared to LF-fed rats (139.6 ± 2.5 vs. 153.0 ± 3.8 mmHg; P < 0.05).

### Effects of HF Diet on Metabolic Parameters

Fasting plasma glucose levels were elevated in HF-fed rats (Table 1). Plasma insulin levels were also higher in HF-fed rats, suggesting the existence of insulin resistance in HF-fed rats. Serum leptin was higher in HF-fed rats. Serum total cholesterol and triglycerides were not different between the two groups of rats.

**Table 1:** Parameters from blood samples of high fat (HF)- and low fat (LF)-fed rats

Parameter	HF-Fed Rats	LF-Fed Rats	P Value
n	6	5	
Fasting plasma glucose (mg/dL)	170.8 ± 21.1	106.6 ± 15.5	< 0.05
Fasting plasma insulin (ng/mL)	2.5 ± 0.1	1.3 ± 0.3	< 0.05
Serum leptin (ng/mL)	9.6 ± 1.2	5.6 ± 0.6	< 0.05
Serum total cholesterol (mg/dL)	78.2 ± 8.5	79.3 ± 7.8	n.s.
Serum triglyceride (mg/dL)	44.3 ± 5.6	49.6 ± 10.0	n.s.

Abbreviations: n.s. = not significant.

### Effects of HF Diet on Urinary Catecholamines

**Figure 3:**
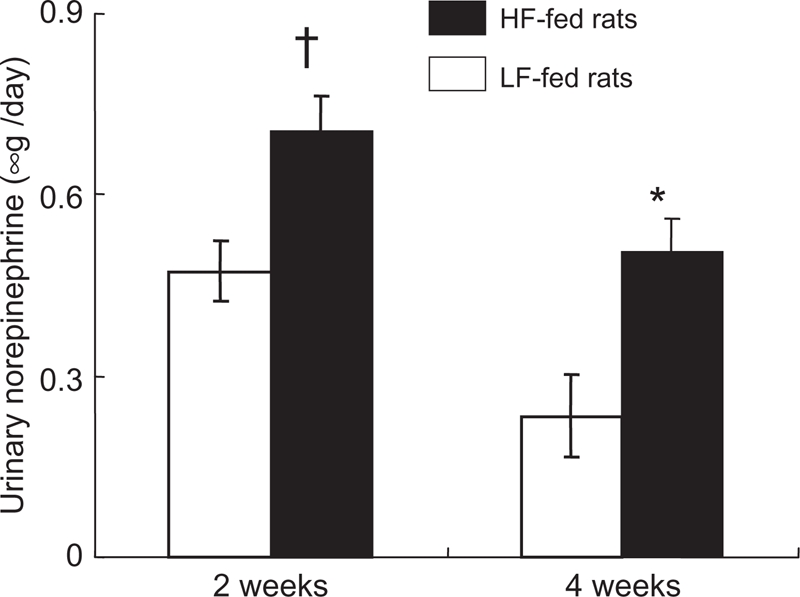
Urinary norepinephrine excretion in high fat (HF)- and low fat (LF)-fed rats (n = 5, respectively). Urinary norepinephrine was significantly increased at 2 and 4 weeks of the treatment in HF-fed rats. Similarly, urinary epinephrine was increased at 4 weeks of the treatment in HF-fed rats. *P < 0.05, ^†^P < 0.01 vs. LF-fed rats, respectively.

Urinary norepinephrine levels were more elevated in HF-fed rats than in LF-fed rats at the early phase of the treatment (2 weeks: P < 0.01; 4 weeks: P < 0.05; Figure 3), suggesting that sympathetic drive existed in HF-fed rats. However, urinary norepinephrine did not differ in HF-fed rats at the late phase of the treatment (6 and 8 weeks: data not shown), probably because the stimulated sympathetic nervous system was masked by high BP-induced, sympatho-inhibition in HF-fed rats.

### Effects of HF Diet on Urinary Protein

**Figure 4:**
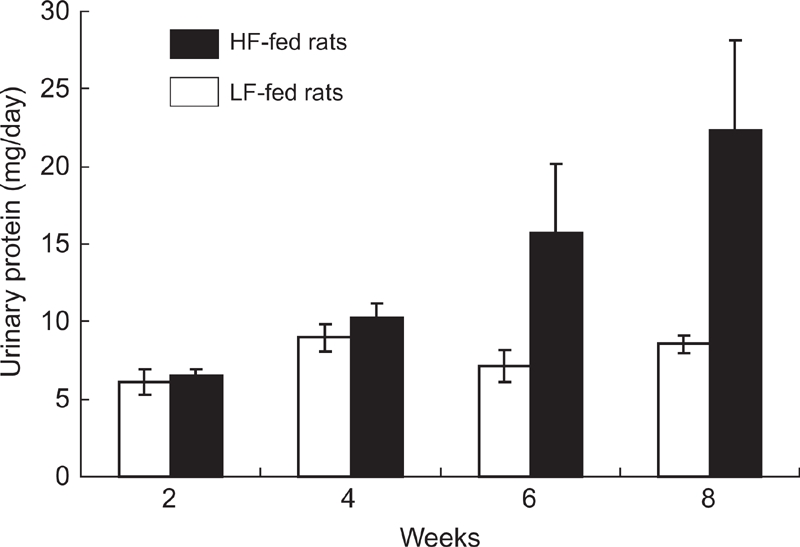
Changes in urinary protein with fat loading. Urinary protein has tendency to be increased at the late phase (6 and 8 weeks) of fat loading in high fat (HF)-fed but not in low fat (LF)-fed rats (n = 5, respectively).

High fat loading increased urinary protein in Dahl S rats (Figure 4). After 8 weeks of treatment, urinary protein of HF-fed rats tended to increase (22.4 ± 5.8 vs. 8.5 ± 0.6 mg/day; P = 0.07).

### Salt Loading after HF or LF Diet

**Figure 5:**
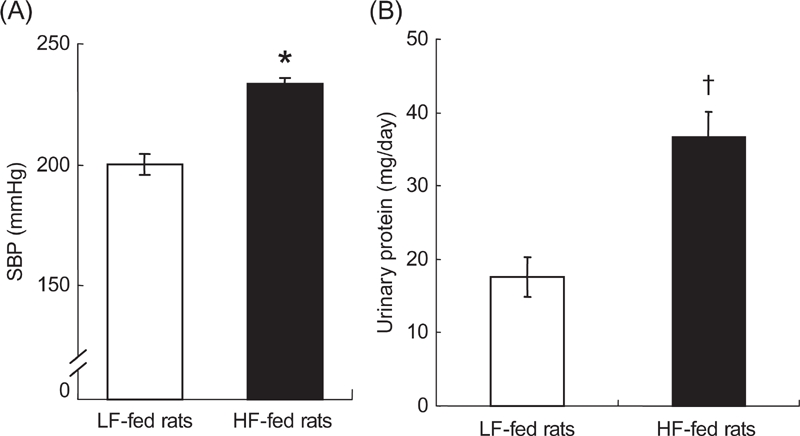
Salt-induced rise in systolic blood pressure (SBP) and urinary protein in high fat (HF)-fed rats (n = 6) and low fat (LF)-fed rats (n = 5). (A) SBP was greater after salt loading in HF-fed rats after 8 weeks of treatment. (B) Urinary protein was also increased in HF-fed rats. *P < 0.05, ^†^P < 0.01 vs. LF-fed rats, respectively.

**Figure 6:**
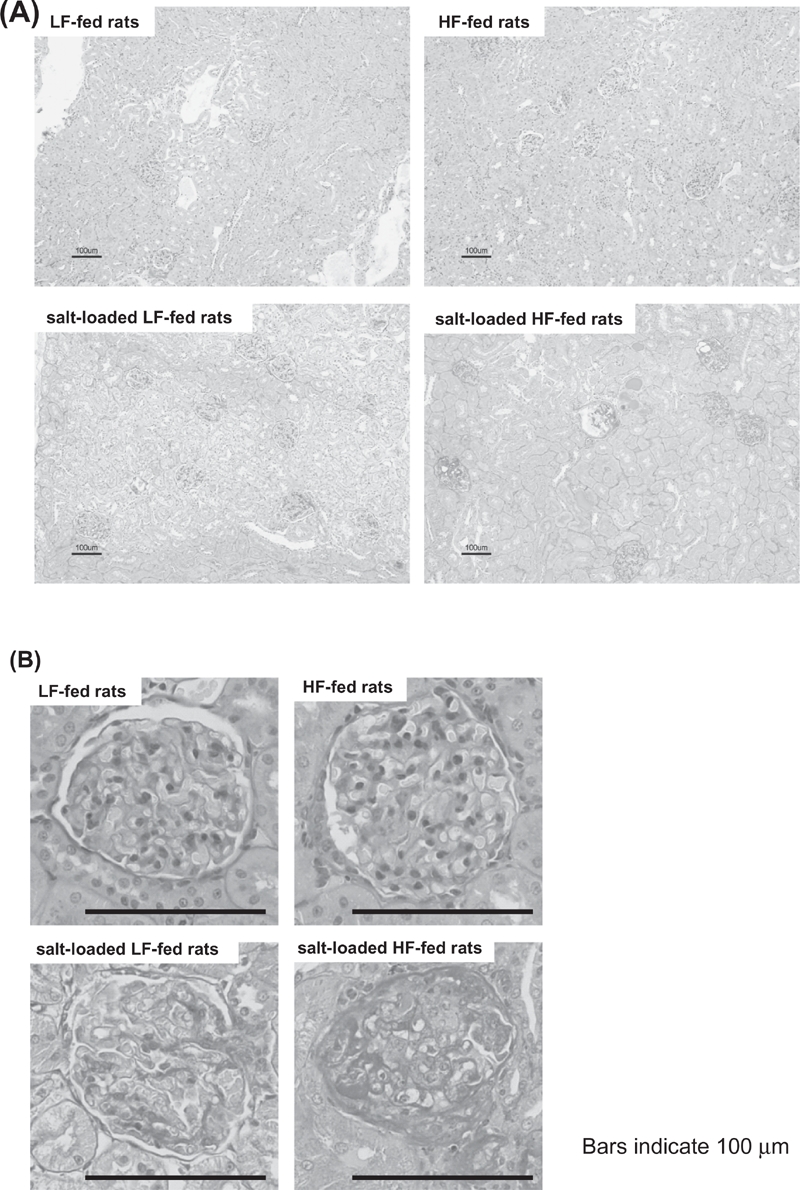
Representative micrographs of periodic acid-Schiff-stained renal (A) and glomerular (B) sections from high fat (HF)-fed rats and low fat (LF)-fed rats before and after salt loading. In salt-fed HF-fed rats, glomerular, tubulointerstitial, and arteriolar damages were more severe. The bar represents 100 μm. The histological findings were confirmed in two kidneys from each group at each point of time.

During salt loading, food intake, and urinary sodium excretion (Table 2) were not different between salt-loaded HF and LF rats. However, SBP was greater in HF-fed rats than in LF-fed rats (8 weeks of the treatment: 220.4 ± 4.4 vs. 195.9 ± 8.8 mmHg; p < 0.05), although salt loading increased SBP in both HF-fed rats and LF-fed rats (Figure 5A). Thus, pressor effect of HF and high salt diet was additive. Urinary protein was also greater in salt-loaded HF-fed rats than in salt-loaded LF-fed rats (36.7±3.4 vs. 17.6±2.7 mg/day; P < 0.01; Figure 5B). As shown in Figure 6, renal histological damage was not apparent in both HF-fed and LF-fed rats before salt loading. However, the glomeruli showed mesangial cell proliferation, matrix overgrowth, and glomerular scleosis and collapse in HF-fed Dahl S rats with salt loading, but the glomerular changes were less in LF-fed rats. The tubulointerstitial damage, interstitial inflammatory cell infiltration, and tubular cast formation together with the hyaline thickening of arteriole were also more apparent in HF-fed Dahl S rats with salt loading. These histological findings were almost the same in two kidneys from each group at each point of time.

**Table 2:** Food intake and urinary sodium excretion of high fat (HF)- and low fat (LF)-fed rats after salt loading was started

		Duration of Salt Loading			
		
	n	2 Weeks	4 Weeks	6 Weeks	8 Weeks
Food Intake (g/day)					
HF-fed rats	6	17.7 ± 0.6	19.6 ± 0.4	17.7 ± 1.0	18.0 ± 1.3
LF-fed rats	5	19.0 ± 0.6	20.7 ± 1.4	20.5 ± 0.3	21.5 ± 2.4
P value		n.s.	n.s.	n.s.	n.s.
Urinary Sodium (mEq/day)					
HF-fed rats	6				16.22 ± 1.02
LF-fed rats	5				15.9 ± 1.32
P value					n.s.

Abbreviations: n.s. = not significant.

## Discussion

In the present study, the HF diet moderately increased SBP in addition to obesity, visceral fat accumulation, and insulin resistance in a salt-sensitive hypertension model, Dahl S rats. Thus, the HF-fed Dahl S rats are a model of diet-induced metabolic syndrome. Interestingly, because visceral fat accumulation preceded body weight gain similarly to a rise in BP, accumulation of visceral fat rather than body weight gain may play an important role in the rise in BP. More importantly, HF intake strengthened not only salt-induced elevation in BP but also salt-induced progress of renal injury.

The HF-fed Dahl S rats showed insulin resistance as suggested by hyperinsulinemia associated with hyperglycemia. Hyperinsulinemia is believed to decrease renal sodium excretion to generate salt-sensitive hypertension (4,16); insulin stimulated the sodium-potassium adenosine triphosphatase pump and the sodium-hydrogen antiporter, two major renal tubular transports for sodium absorption. Although defects on the level of insulin receptor substrates (IRS)-1 may underlie some forms of insulin resistance, recent reports indicated that sodium retention, facilitated by hyperinsulinemia through the IRS-1-independent pathway, could be an important factor in pathogenesis of hypertension in insulin resistance (17). Our data suggest that in Dahl S rats, a model of salt-sensitive hypertension, HF diet accentuates potentially existing insulin resistance (6), which strengthens a salt-induced rise in BP. Salt loading not only made clear that Dahl S rats have potential insulin resistance (5) but also increased insulin resistance even in SD rats without BP elevation (18). Therefore, salt loading itself might have a metabolic effect, which is more apparent in Dahl S rats possibly because of their genetic predisposition. This hypothesis makes it possible to explain enhanced “fat sensitivity of BP” as well as increased salt sensitivity of BP in Dahl S rats. Only 2 weeks of fat loading increased BP in Dahl S rats, although 2-month-HF loading had not affected BP in Fischer rats (2).

In addition, hyperinsulinemia is believed to cause sympatho-excitation (19), which may also contribute to increased BP in HF-fed Dahl S rats. In the present study, urinary norepinephrine was increased in the early phase of fat loading. Actually, calorie restriction reduced BP in obese hypertension by the improvement of autonomic nerve activation (20). Reversely, sympathetic stimulation may enhance insulin resistance (21). Thus, sympathetic overactivity and insulin resistance may stimulate each other, both of which may importantly contribute to a rise in BP. Moreover, additional factors, such as leptin (22) and oxidative stress (23,24), may contribute to sympathetic drive and hypertension in obesity. Indeed, serum leptin was increased with fat loading in Dahl S rats. In addition, our recent studies suggested that brain oxidative stress causes the central sympathetic nerve stimulation and the resultant hypertension in both salt-sensitive (25) and obese hypertension (26).

In Dahl S rats, a HF diet tended to increase urinary protein without renal histological changes. Even though the renal damaging effect of a HF diet was only marginal, a HF diet accelerated salt-induced proteinuria and renal histological aggravation. These may not be explained by a mild BP rise alone; urinary protein increased approximately two times and histological damages were apparent whereas BP increased only about 1.1 times. Indeed, salt loading extremely damaged the kidney in a metabolic syndrome model, SHR/NDmcr-cp, without an increase in BP (13). Hyperinsulinemia may be one of the candidates to accelerate salt-induced progression of renal damage. For example, insulin stimulated the proliferation of renal cells (27) and produced growth factors such as insulin-like growth factor (IGF)-1 (28) and transforming growth factor (TGF)-beta (29). Moreover, insulin resistance and hyperinsulinemia increased oxidative stress, which has been implicated in the progress of nephropathy (11,13,30). Insulin resiatnce should be intensified by salt loading, which may further progress renal damage (5). Moreover, sympathetic drive may also facilitate damaging process of the kidney (31,32). Although a precise mechanism should be clarified in further experiments, it should be important that fat excess accelerated salt-induced renal injury.

In conclusion, fat loading stimulated salt-induced harmful effects, such as BP rise and renal damage, possibly through insulin resistance and sympathetic overactivity in Dahl S rats, suggesting that a combined disturbance of lifestyle (excess of both fat and salt) may cause more severe hypertension and/or renal damage especially in the biotype of salt-sensitive hypertension.
